# One toolkit to bring them all, and *in silico* analyze them

**DOI:** 10.1002/ctd2.194

**Published:** 2023-04-21

**Authors:** Chennan Li, Anna Baj, Adam G. Sowalsky

**Affiliations:** Laboratory of Genitourinary Cancer Pathogenesis, Center for Cancer Research, National Cancer Institute, Bethesda, Maryland, USA

**Keywords:** bioinformatics, cancer, circulating tumor DNA, genomics

Circulating tumor DNA (ctDNA) is a class of short tumor-derived DNA molecules that are typically detected in body fluids including plasma. Recent evidence suggests that certain molecular characteristics of ctDNA associated with various aspects of cancer transcriptome and ctDNA abundance can inform disease burden.^1^ These findings have shown the promise of utilizing liquid biopsy for tracing disease progression and guiding clinical decisions. Despite the increasing advances in ctDNA research and the diverse computational workflows developed to support such research, computational toolsets that tackle multiple critical questions at once are lacking yet are highly needed by the science community. To address this need, Li et al. recently developed a comprehensive toolkit for assessing cancer transcriptomic dynamics using the whole-genome sequencing (WGS) data of cell free DNA (cfDNA), which contains DNA of normal cells and ctDNA.^2^ This tool, named Integrated analysis toolkit for whole-genome-wide features of cfDNA (INAC), carries out multiple functions including generating whole-genome copy number profiles, estimating gene expression using fragment-based analyses, and identifying disease predictive features using machine learning (see Figure 1).

Human cancers often undergo massive chromosomal rearrangements, resulting in aneuploidy that is detected through somatic copy number profiling. Depending on how much ctDNA species are captured (also known as the tumor or ctDNA fraction), the somatic copy number profile of a cfDNA sample can reflect tumor aneuploidy to a certain degree. In cases where multiple diseases co-occur in an individual, a mixture of the copy number alterations of both diseases is likely observed when analyzing cfDNA. Therefore, the copy number profile of a cfDNA sample may inform tumor burden over the time and disease classification, which is evaluated by the INAC_CNV module (see Table 1).

In addition, multiple analyses of ctDNA fragmentation patterns that are provided by the toolkit are essential to transcriptomic modeling. The ctDNA species identified in plasma originate from fragmented DNA that remains bound by histones but is released from tumor cells in its nucleosome-bound form.^9^ Therefore, the distribution of ctDNA reads mapped to a genomic locus can be used to infer chromatin accessibility. In this context, open chromatin is associated with a higher percentage of short ctDNA fragments (or a high short/long fragment ratio) and a lower read abundance at promoters may indicate active gene expression, and vice versa^10^; see Figure 1). To enable the assessment of expression rate, INAC calculates the long-to-short fragment ratio and nucleosome-depleted region sequence depths at various regions flanking transcriptional start sites (TSSs) using the INAC_TSS module. Additionally, a recently published article described promoter fragmentation entropy (PFE) as another measure of gene expression state.^5^ The underlying basis is that highly expressed genes are associated with an open chromatin state and thus more diverse histone binding pattern, which is recognized as a greater variety of ctDNA fragment sizes with a high PFE (see Figure 1). The algorithm that assesses this fragmentation feature is also included in the INAC for evaluating expression states as INAC_PFE.

Finally, one potential clinical application of ctDNA analysis is using its various molecular characteristics for predicting disease states and clinical outcome. Li et al. showed that ctDNA fragmentation patterns at TSSs could predict cancer and normal tissues better than other tested features using different machine learning (ML) methods. It is possible that additional fragmentation features that can be captured by the toolkit and have not yet been explored may be better associated with cancers, or a specific subtype of a cancer using the provided ML algorithm. Clearly, the accessibility to the toolset reveals new possibilities of identifying potential predictive features for a given disease.

For researchers who are new to computational analysis of ctDNA, the toolkit described by Li et al. has particular benefits. The entire script was written in R, which is commonly used by biological scientists and therefore allows for easier troubleshooting and additional customization and code modifications. For instance, users can compare fragments with any sizes of interest and evaluate the fragmentation pattern at a specific set of genomic loci such as enhancers or non-coding RNAs. Gathering this information may foster new hypothesis testing to generate novel research ideas. For example, if a researcher wished to test whether a chromatin state is altered at genomic locations enriched for a transcriptional motif or regulatory sequence over the course of a therapy or a metastatic event, this use can be quickly assessed using INAC.

A limitation of this toolset lies in its intentional design to assess various fragmentomic properties of ctDNA and not other recently discovered molecular features, such as base- or motif-level information for additional functional implications.^11,8^ Unfortunately, INAC does not support ML modeling for distinguishing various disease types using ctDNA features, which may easily be addressed. Once additional molecular patterns of ctDNA are described that implicate other essential biological functions, further expanding this WGS-based toolkit would be beneficial for the rising ctDNA community. The INAC toolkit as a whole has significant future potential in aiding our understanding of cancer biology via the analysis of ctDNA and its assessment of clinical utility.

## Figures and Tables

**FIGURE 1:**
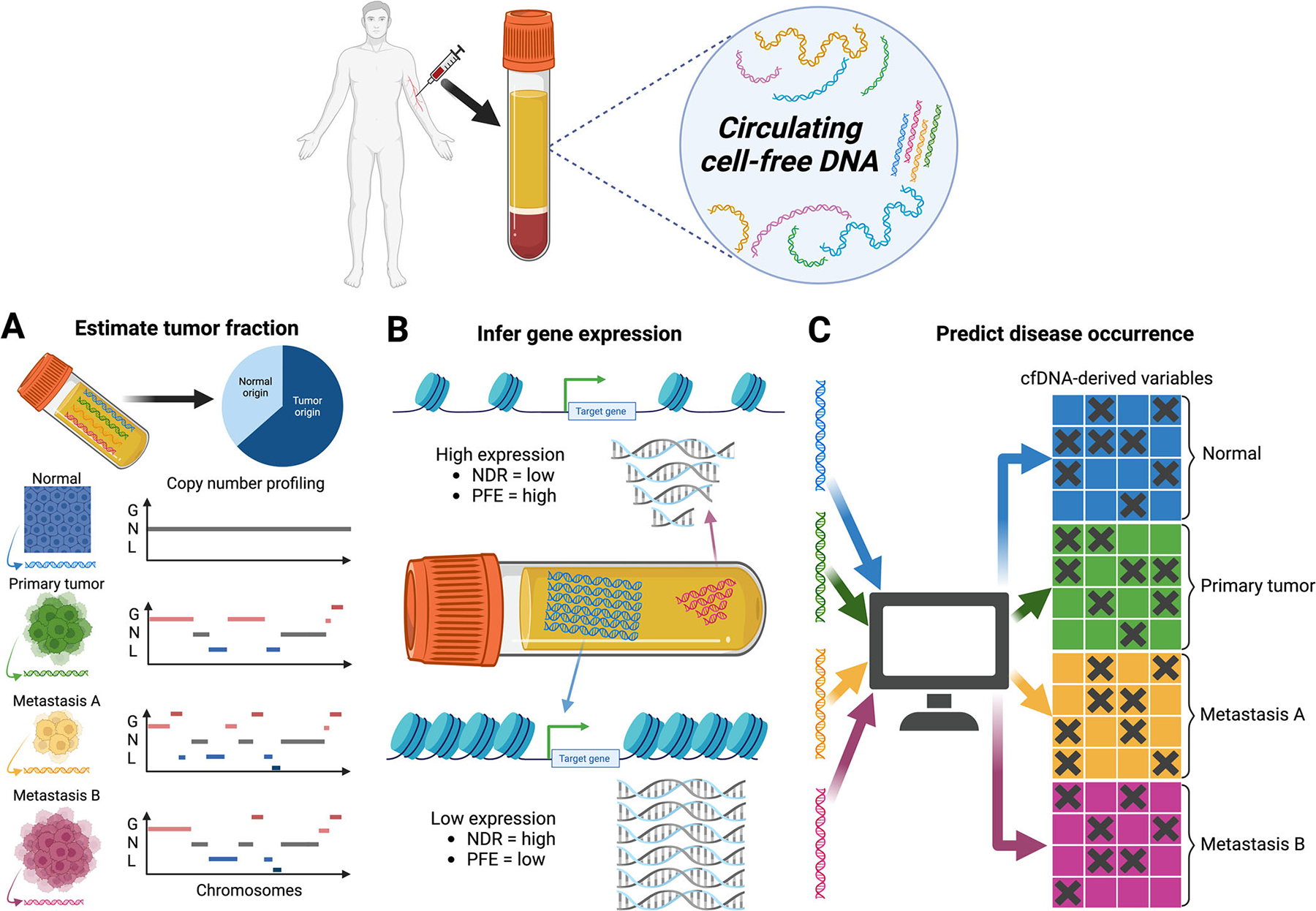
Cell free DNA (cfDNA) contains multiple information that informs the biological basis of a disease and predicts disease occurrence. (A) Genomic features of cfDNA such as copy number alterations (CNAs) can be used for estimating tumor fraction and classifying diseases. The diseased and normal tissues carry distinct copy number profiles (G, gain; N, neutral; L, loss), which are used for determining the proportion of each disease in a cfDNA sample. (B) The size distribution of ctDNA fragments can inform gene expression rate. Actively transcribed genes are associated with a low coverage of nucleosome-depleted regions (NDR) and a high promoter fragmentation entropy (PFE). (C) Variables associated with cfDNA such as CNAs may be used for predicting the existence of diseases using machine learning algorithms.

**TABLE 1:** Usage of cfDNA-derived variables in clinical research that are supported by current toolkits and INAC modules.

Molecular features	Potential usage	INAC module capable of the analysis	Existing tools	References
Somatic variant frequency	Predict tumor fraction and estimate subclonal fraction	-	Somatic variant callers such as Mutect2	3
Copy number profile	Predict tumor fraction and subclonal prevalence	INAC_CNV	Copy number profilers such as Titan-CNV	4
Fragment lengths	Examine sampling quality	INAC_QC/FR	Pre-WGS sample QC or computational tools for coverage and insert quality analyses	-
Sequence coverage at nucleosome-depleted regions (NDR)	Estimate gene expression activity	INAC_TSS	Epigenetic expression inference from cell-free DNA-sequencing (EPIC-seq)	5
Promoter fragmentation entropy (PFE)	Estimate gene expression activity	INAC_PFE	EPIC-seq	5
Transcriptional motifs	Predict transcriptional activity	-	Griffin	6
Somatic variants/CNAs	Model clonal heterogeneity	-	ctDNA-based subclonal reconstruction	7
Any of the above features	Classify disease or predict therapy outcomes	INAC_ML	Machine learning tools	8

Abbreviation: CNAs, copy number alterations.
